# Efficient Oxidation of Cyclohexane over Bulk Nickel Oxide under Mild Conditions

**DOI:** 10.3390/molecules27103145

**Published:** 2022-05-14

**Authors:** Reem S. Alnefaie, Mohamed Abboud, Abdullah Alhanash, Mohamed S. Hamdy

**Affiliations:** Catalysis Research Group, Chemistry Department, College of Science, King Khalid University, Abha 61413, Saudi Arabia; 438800026@kku.edu.sa (R.S.A.); alhnsh@kku.edu.sa (A.A.); mhsaad@kku.edu.sa (M.S.H.)

**Keywords:** bulk nickel oxide, cyclohexane oxidation, *m*-CPBA, KA oil, mild conditions

## Abstract

Nickel oxide powder was prepared by simple calcination of nickel nitrate hexahydrate at 500 °C for 5 h and used as a catalyst for the oxidation of cyclohexane to produce the cyclohexanone and cyclohexanol—KA oil. Molecular oxygen (O_2_), hydrogen peroxide (H_2_O_2_), t-butyl hydrogen peroxide (TBHP) and *meta*-chloroperoxybenzoic acid (*m*-CPBA) were evaluated as oxidizing agents under different conditions. *m*-CPBA exhibited higher catalytic activity compared to other oxidants. Using 1.5 equivalent of *m*-CPBA as an oxygen donor agent for 24 h at 70 °C, in acetonitrile as a solvent, NiO powder showed exceptional catalytic activity for the oxidation of cyclohexane to produce KA oil. Compared to different catalytic systems reported in the literature, for the first time, about 85% of cyclohexane was converted to products, with 99% KA oil selectivity, including around 87% and 13% selectivity toward cyclohexanone and cyclohexanol, respectively. The reusability of NiO catalyst was also investigated. During four successive cycles, the conversion of cyclohexane and the selectivity toward cyclohexanone were decreased progressively to 63% and 60%, respectively, while the selectivity toward cyclohexanol was increased gradually to 40%.

## 1. Introduction

**Figure 1:**
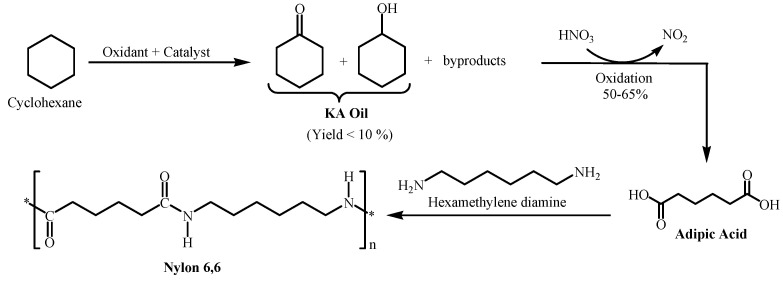
Synthesis pathway of nylon 6,6 from cyclohexane.

The oxidation of cyclohexane is an important industrial chemical reaction. The oxidation of cyclohexane affords cyclohexanol and cyclohexanone, which are together known as ketone–alcohol (KA) oil, the main feedstock for the production of nylon 6,6 fibers (Figure 1). The further oxidation of KA oil by nitric acid led to the formation of adipic acid, which is a key monomer for the preparation of very important polymers such as nylon 6,6 [1]. In addition, adipic acid is an important synthetic intermediate in the chemical industry [2,3].

The current industrial process for the oxidation of cyclohexane to produce KA oil involves the utilization of cobalt or manganese salts as homogenous catalysts. The operating conditions consist of high temperature (150–160 °C) and high pressure (10–20 atm) of air or molecular oxygen (O_2_) due to the stable nature of the cyclohexane. However, since the desired products, cyclohexanol and cyclohexanone, are less stable than the starting material, cyclohexane, many by-products are formed during the oxidation of cyclohexane at high temperature and pressure. Thus, at high conversion of cyclohexane, the KA oil can be formed with low selectivity, which makes the purification of KA oil difficult with a high-cost process, in addition to the major issue of the regeneration and reutilization of the homogeneous catalysts. Therefore, the commercial processes usually operate at a low cyclohexane conversion of about 4–6% to maintain the high selectivity of KA oil at 70–85% [4].

To improve the process for the oxidation of cyclohexane, the heterogeneous catalysts have been considered as good alternatives to improve the cyclohexane conversion while maintaining high KA oil selectivity and minimizing the catalyst utilization and separation costs.

Therefore, many heterogeneous catalytic systems have been developed during the last two decades. Various supported metal catalysts were prepared, such as metal transition Au, Ti, Ag, Co, Mo, Fe, Mn, Cr, and V, and even lanthanides such as La, Ce, Sm, Dy, Y, and Gd, using different type of supports such as silica, alumina, zeolites, graphite, and aluminophosphates (AlPo) [5]. Air was the preferred oxidant due to its low cost, along with other oxidants such as molecular oxygen (O_2_), hydrogen peroxide (H_2_O_2_), and t-butyl hydrogen peroxide (TBHP). However, the utilization of H_2_O_2_ and TBHP is not industrially viable due to their high cost compared to air and O_2_. Thus, higher attention has been paid to the liquid phase process using O_2_ as an oxidation agent.

The high dispersion of some transition metals in high surface-area supports, such as gold nanoparticles in mesoporous silica, exhibited relatively effective oxidation of cyclohexane. Xu et al. [4] reported the utilization of silica-supported gold catalyst doped with titania under air at high temperature (150 °C) and high pressure (1.5 MPa) to convert the cyclohexane to KA oil. However, the highest conversion was 9.2% with 82.6 selectivity. Similar results (10–13% conversion, 84–87% selectivity) were obtained by Xu and coworkers when gold was supported on alumina (Au/Al_2_O_3_) under similar conditions, using the molecular oxygen as an oxidant [6]. Better results (16.6% conversion, 92.4% selectivity) were obtained with highly dispersed gold nanoparticles on functionalized SBA-15 mesoporous silica, using molecular oxygen under similar conditions (150 °C, 1 MPa) [7]. Similar results (16.9% conversion, 93% selectivity) were obtained by Wang et al. [8] when bismuth-containing SBA-15 (Bi-SBA-15) was used as catalyst, with molecular oxygen as oxygen donor.

However, when supported gold was used in mild conditions, both conversion and selectivity decreased. For example, when Enache et al. used graphite-supported gold under mild conditions (70 °C, 1 atm) the conversion (2–7%) and selectivity (10–23%) were very low [9]. However, all of these catalytic systems still suffer from low cyclohexane conversion, expensive and complicated synthesis of the catalyst, and high-cost processes. Hence, the selective oxidation of cyclohexane with high conversion in mild conditions is still a challenge. Therefore, there is still great interest among industry and academia for the development of simple, low-cost, reusable and efficient heterogeneous catalytic systems.

One of the most promising transition metals is nickel. Due to its relative abundance, nickel is more cost-effective than most metals as a catalyst [10]. Numerous research efforts have been recently devoted to the preparation of nickel particles with tailored features, because of their unique electronic, optical, and mechanical properties and their widespread potential applications in many fields including catalysis, electronics, optoelectronics, adsorption of dyes from industrial water, development of supercapacitors, fabrication of dye-sensitized solar cells and sensors, and biomedical applications [11,12,13,14,15,16,17,18,19,20,21,22,23,24,25,26,27,28,29,30,31,32].

Due to their unique magnetic, chemical, and physical properties, we believe that Ni particles will gain more attention in future in various technological fields such as catalysis, battery manufacture, dye-sensitized solar cells, enhanced pseudo-capacitance, and drug delivery.

Recently, Ni-based heterogeneous catalysts have been employed for various organic transformations such as hydrogenation reactions of aromatics [33], oxidation of hydrocarbons [34,35,36], production of synthesis gas [37], steam reforming [38], methanation [39], isomerization of hydrocarbons [40], hydrocracking [41], etc. [42,43,44]. Ni is considered as a promising catalyst because it is eco-friendly, inexpensive, easy to prepare, and easily recoverable and recyclable. 

In this work, we report a very simple and highly efficient nickel-based heterogeneous catalytic system for the oxidation of cyclohexane to KA oil. Surprisingly, the utilization of bulk nickel oxide as a catalyst with *meta*-chloroperoxybenzoic acid (*m*-CPBA) as oxidant in mild conditions allowed us to quantitively convert cyclohexane to KA oil with 99% selectivity.

## 2. Results

### 2.1. Characterization of Bulk NiO

The prepared bulk NiO was characterized by SEM, EDX, XRD and TGA.

**Figure 2:**
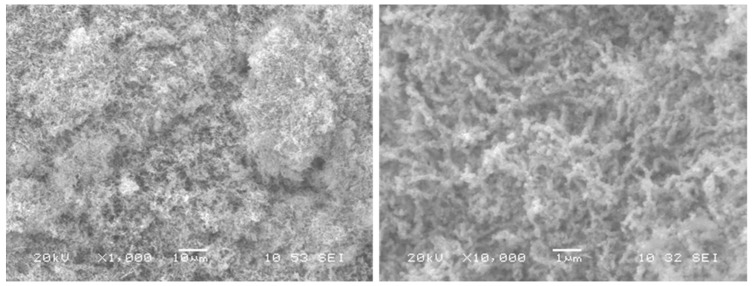
SEM pictures of NiO powder.

**Figure 3:**
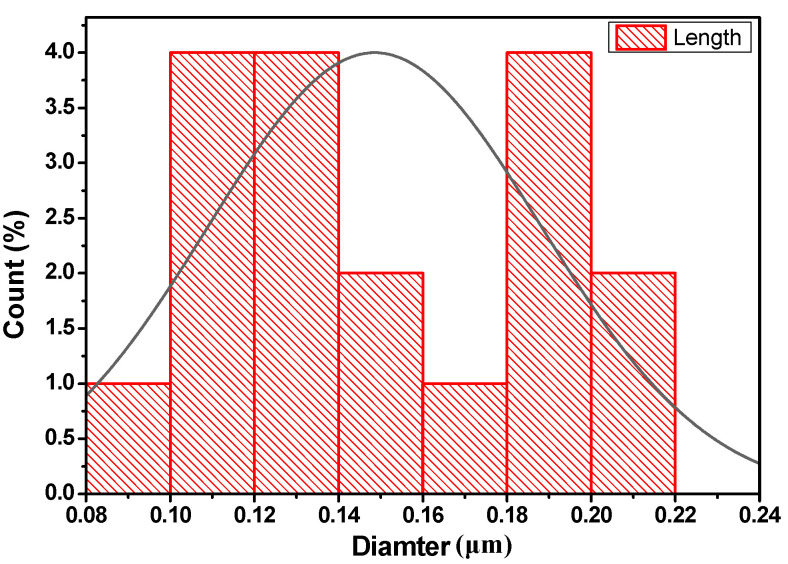
Particle diameter distribution in NiO catalyst.

SEM images (Figure 2) show aggregated microparticles of nickel oxide particles forming worm-like shapes. The particle size distribution was measured using ImageJ software, and the obtained results are presented in Figure 3. The average NiO particles size was found to be around 148 nm.

**Figure 4:**
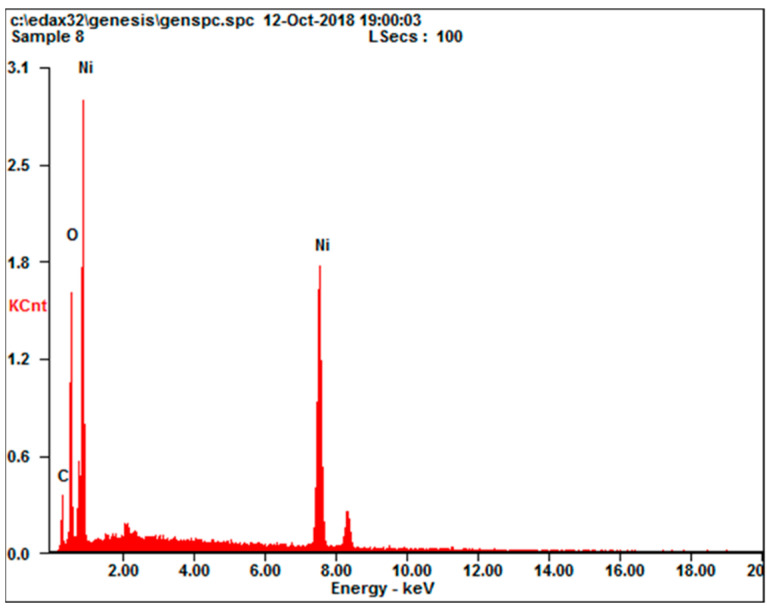
EDX spectra of NiO powder.

The prepared NiO was also studied by EDX. Figure 4 shows the EDX spectrum of NiO particles. Ni and O peaks were clearly observed, and the [Ni]/[O] ratio was 1.13, which is consistent with NiO molecular structure.

**Figure 5:**
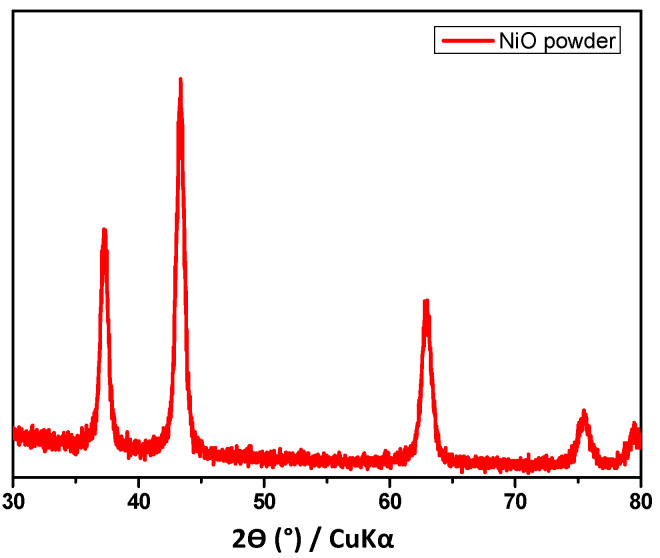
XRD pattern of NiO powder.

Powder XRD analysis was used to identify the synthesized NiO powder (Figure 5). The XRD pattern shows the principal peaks of NiO, which were observed at 2Ɵ = 37.31°, 43.41°, 62.87°, 75.53°, and 79.46° and assigned to the (111), (200), (220), (311), and (222) planes, respectively [45]. This confirmed the formation of pure NiO particles [46,47].

**Figure 6:**
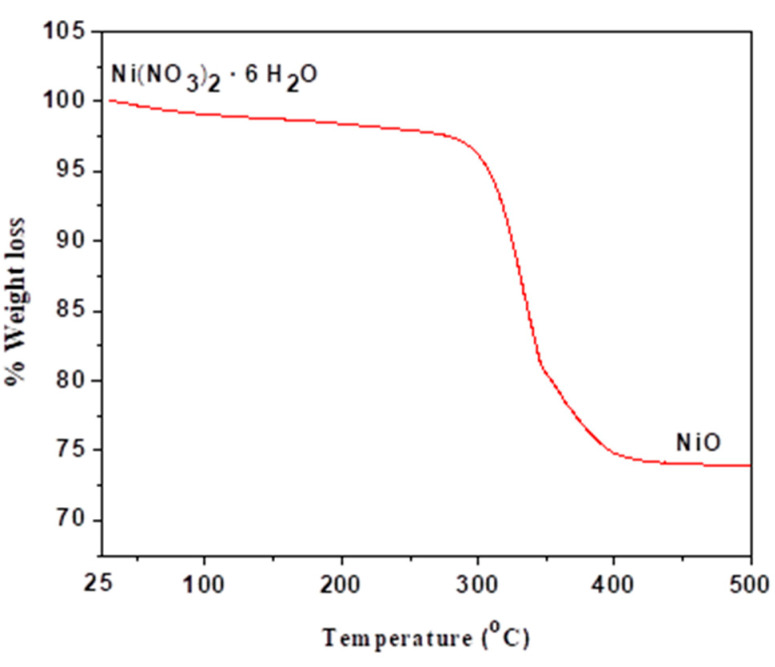
TGA thermogram of the decomposition of nickel nitrate hexahydrate under air.

Figure 6 illustrates the thermal decomposition patterns of nickel nitrate hexahydrate precursors under air. The thermogram of Ni(NO_3_)_2_·6H_2_O can be divided into three segments. The first region from 25 °C to 250 °C with low weight loss of about 2 wt.% corresponds to the elimination of residual solvents and physically absorbed water. The second segment from 250 °C to 440 °C, with rapid weight loss of about 28%, is related to water separation and decomposition of Ni(NO_3_)_2_ and formation of NiO.

### 2.2. The Oxidation of Cyclohexane over NiO Powder

#### 2.2.1. Effect of Oxidant

**Figure 7:**
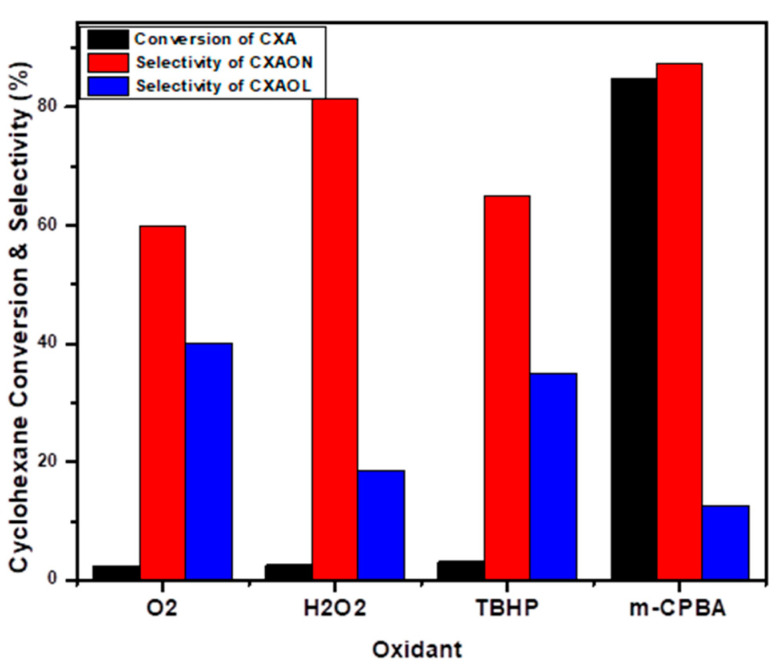
Oxidation of cyclohexane using different types of oxidants.

The selective oxidation of cyclohexane in the presence of NiO as a catalyst was investigated together with the liquid-phase oxidation by using different oxidants at 70 °C for 24 h, and the obtained results are presented in Figure 7. When molecular oxygen was used as an oxidant, only 2.4% of cyclohexane was converted to the oxidized products: cyclohexanone (60%) and cyclohexanol (40%). The use of liquid-phase oxidation in the presence of H_2_O_2_ and TBHP did not improve the conversion of cyclohexane, and only 2.5% and 3.1% of KA oil was obtained, respectively. A dramatic increase in cyclohexane oxidation was obtained in the liquid-phase system when *m*-CBPA was applied as an oxidant; 84.8% of cyclohexane was oxidized to 99% KA oil, with 87.4% and 12.6% selectivity towards cyclohexanone and cyclohexanol, respectively. The obtained results clearly show that the catalytic activity of NiO can be severely improved by using a suitable oxidant such as *m*-CBPA.

#### 2.2.2. Effect of Reaction Temperature

**Figure 8:**
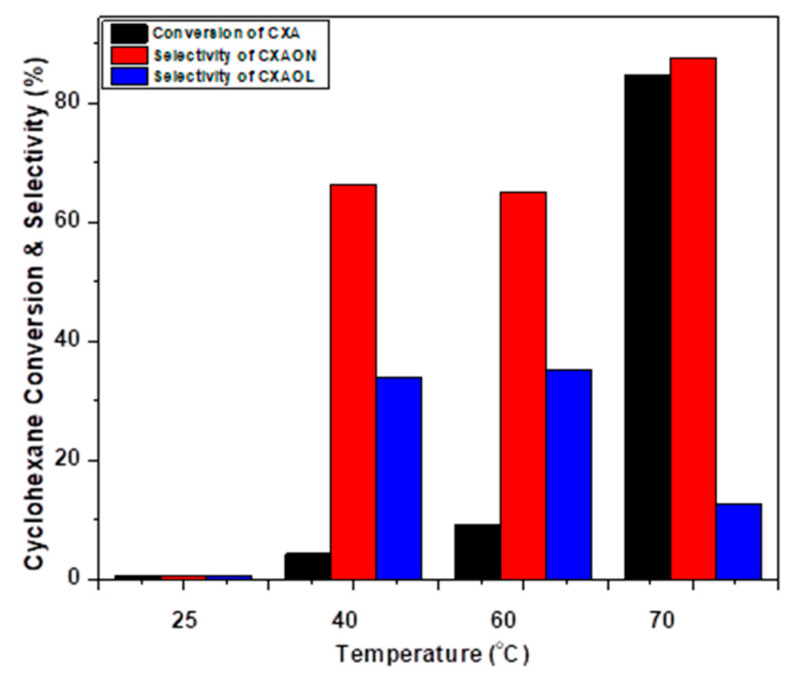
The oxidation of cyclohexane by *m*-CPBA for 24 h using different temperatures.

The selective oxidation of cyclohexane by using NiO powder as a catalyst and *m*-CBPA as an oxidant was investigated under different applied temperatures: 25, 40, 60 and 70 °C. The obtained results are presented in Figure 8, which shows that an almost negligible amount of cyclohexane was oxidized at room temperature, and less than 5% conversion was obtained at 40 °C or 60 °C. However, the best results were observed at 70 °C, with 84.8% cyclohexane conversion, with 99% KA selectivity and 87.4% selectivity towards cyclohexanone. 

#### 2.2.3. Effect of Reaction Time

**Figure 9:**
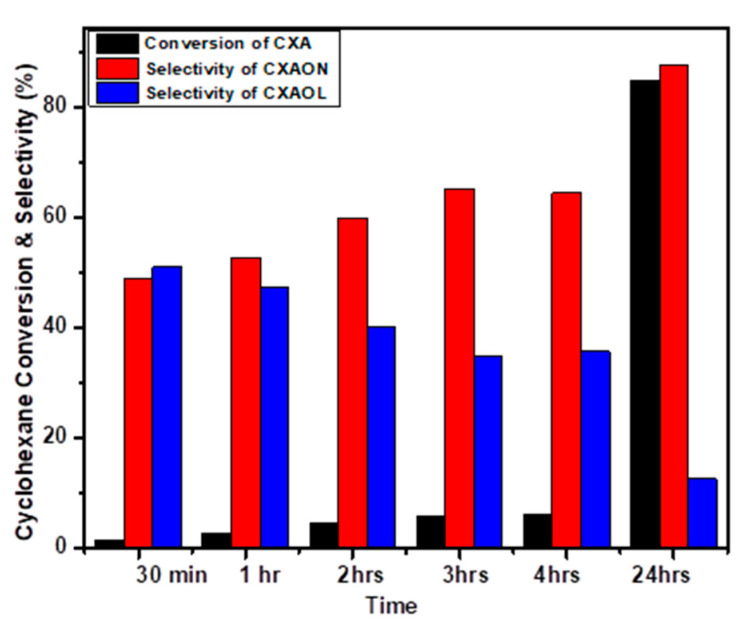
Effect of reaction time on the oxidation of cyclohexane over NiO powder, using *m*-CPBA, T = 70 °C, time: 30 min–24 h.

The selective oxidation of cyclohexane using NiO as a catalyst and *m*-CPBA as an oxidant was studied at different reaction times at 70 °C. The obtained results are plotted in Figure 9. After 4 h of the reaction, less than 5% conversion of cyclohexane was obtained. More importantly, the selectivity of cyclohexanol was found to be decreased, while the selectivity of cyclohexanone increased, which means that *m*-CPBA could also oxidize cyclohexanol to cyclohexanone. The obtained results confirm the relatively long life of the oxidant with high ability to oxidize cyclohexane during the entire reaction time. Moreover, *m*-CPBA was not only active in the oxidation of cyclohexane, but also more selective towards cyclohexanone. The best results were obtained after 24 h with around 85% conversion, 99% KA oil selectivity, with 87% of cyclohexanone and 12% of cyclohexanol.

#### 2.2.4. Effect of Catalyst Amount

**Figure 10:**
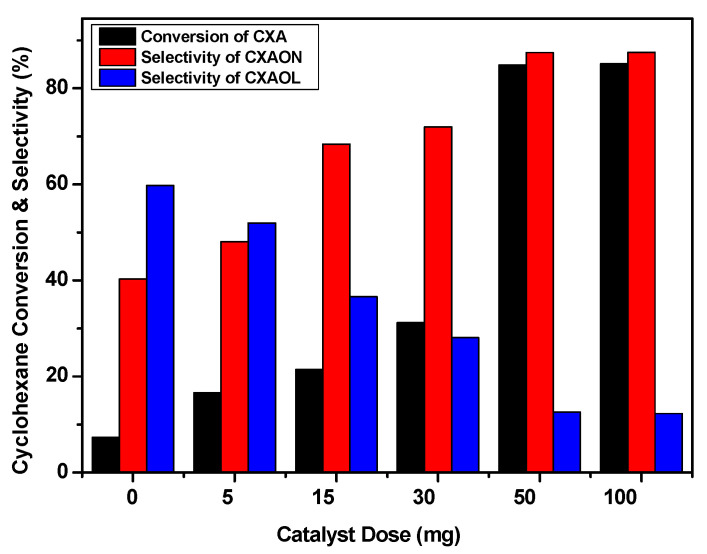
Catalyst dose effect. Reaction conditions: Catalyst is bulk NiO, *m*-CPBA, T = 70 °C, 24 h.

The catalyst amount was optimized in order to achieve the best substrate/catalyst ratio. Several amounts of NiO were used in separate experiments to oxidize cyclohexane at 70 °C for 24 h in the presence of 1.5 eq of *m*-CPBA as an oxidant. The obtained results are presented in Figure 10. The conversion of cyclohexane was found to be increased by increasing the amount of NiO catalyst, while the amount of the produced cyclohexanol was found to be decreased. The best result was obtained when 50 mg of bulk NiO was applied, and no significant change was observed when the NiO dose was increased to 100 mg. It should be noted that the reaction proceeded without NiO catalyst and in the presence of *m*-CPBA. However, the conversion of cyclohexane to KA oil was very low, about 7.3%, which shows the importance of the catalyst.

### 2.3. Catalyst Recycling

**Figure 11:**
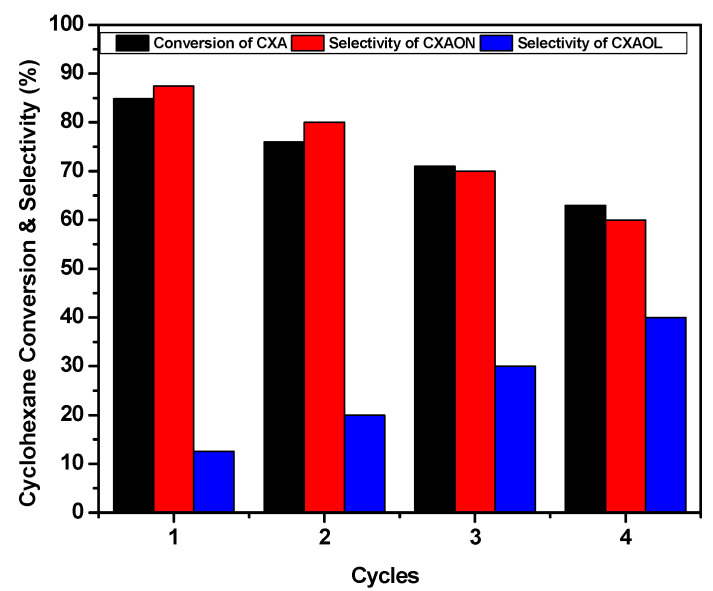
Reusability of NiO powder as a catalyst in the oxidation of cyclohexane with *m*-CPBA. Conditions: catalyst: 50 mg, *m*-CPBA (1.5 eq), time: 24 h, T = 70 °C, 1 atm.

To investigate the stability and reusability of NiO catalyst for the oxidation of cyclohexane in the reaction conditions, NiO powder was recycled and reused under the same conditions mentioned above for four consecutive runs, and the obtained results are presented in Figure 11. After cycle one of the oxidation reaction of cyclohexane, the NiO catalyst was separated by filtration using a centrifuge. Then, the recycled NiO was washed three times with chloroform to remove the reactants, products, and the *meta*-chlorobenzoic acid (*m*-CBA) that were formed after the degradation of the *m*-CPBA. Then, the obtained NiO powder was dried in an oven at 100 °C for around 15 h. After this treatment, the NiO powder was ready to be reused in the following cycle. This treatment was repeated after each cycle, and the final products were detected by GC to calculate the CXE conversion and the product selectivity according to Equations (1)–(3) (above).

The obtained results show a slight decrease in the activity during four successive runs. The cyclohexane conversion was decreased from 85% to 63% with constant KA oil selectivity (99%), and the selectivity of cyclohexanone and cyclohexanol was decreased from 87% and 12% to 60% and 39%, respectively. This decrease in the conversion of cyclohexane was probably due to diminution of NiO amounts during the four cycles. The amount of NiO can be decreased by loss during the recycling and washing process, and also by the degradation (leaching) of NiO particles in the reaction acidic medium.

## 3. Materials and Methods

### 3.1. Materials 

Nickel (II) nitrate hexahydrate (≥98.5%), cyclohexane (ACS reagent, ≥99%), cyclohexanone (analytical standard), cyclohexanol (analytical standard), *meta*-chloroperoxybenzoic acid (*m*-CPBA) (≤77%), t-butyl hydrogen peroxide (TBHP) 5.0–6.0 M in decane, hydrogen peroxide (H_2_O_2_) 50 wt.% in H_2_O, stabilized, molecular oxygen O_2_ cylinder supplied by Southern Gases, acetonitrile (≥99.5%), n-hexane (≥98%), chloroform (≥99.5%), magnesium sulphate (anhydrous, reagent grade, ≥97%) were purchased from Sigma Aldrich. All reagents were of analytical grade and used without further purification.

### 3.2. Methods

#### 3.2.1. Synthesis of NiO Powder

The bulk NiO particles were prepared via the thermal treatment of nickel (II) nitrate hexahydrate. In a typical synthesis reported previously [30], 5 g of nickel nitrate was heated under static conditions in a muffle furnace at 500 °C for 5 h with a heating ramp of 5 °C min^−1^. The obtained material was a black-gray powder.

#### 3.2.2. Characterization of NiO Powder

The prepared NiO powder was characterized by X-ray diffraction (XRD), scanning electron microscopy (SEM), energy dispersive X-ray (EDX), and thermogravimetric analysis (TGA).

The morphology of the obtained NiO powder was observed using SEM Philips EM 300 (Siemens Autoscan, Munchen, Germany). The X-ray diffraction pattern was measured on a Shimadzu Lab-XRD–6000 with CuKα radiation and a secondary monochromator. TGA was investigated under air conditions using a Shimadzu thermogravimetric analyzer operating at a rate of 50 mL min^−1^ of air. In this process, a 20.0 mg sample was submitted in a platinum crucible and heated at 15 K min^−1^ from 30 to 900 °C.

The epoxidation reaction was monitored by a Shimadzu GC-17A gas chromatograph (GC) equipped with flam ionization detector and RTX-5 column, 30 m × 0.25 mm, 1 µm film thickness. Helium was used as the carrier gas at a flow rate of 0.6 mL/min. Samples were withdrawn from the reaction mixture periodically. Injection volume was 1 µL, and total flow was 100 mL/min.

### 3.3. Oxidation of Cyclohexane over Bulk NiO

In order to determine the optimal conditions for the oxidation of cyclohexane over bulk NiO, such as the temperature, reaction time, catalyst dose and the best oxidant, the extreme conditions of the temperature (70 °C), reaction time (24 h) and catalyst amount as used in the literature [5] were used as the starting point in this study to determine the best oxidant. Different oxygen donors were used in this study, including H_2_O_2_, O_2_, TBHP and *m*-CPBA.

The most promising results were obtained with *m*-CPBA. Therefore, other parameters were also investigated using *m*-CPBA as the oxidant, such as the effect of catalyst dose, temperature, and reaction time. The following are the experimental conditions used for each oxidant. Each reaction was run twice. The dodecane was used as internal reference. The oxidation reaction was monitored by GC, and the average conversion and selectivity are presented. 

#### 3.3.1. Molecular Oxygen (O_2_)

The oxidation of cyclohexane over bulk NiO using molecular O_2_ was performed in a high-pressure reactor vessel. A 100 mg amount of the bulk NiO, 20 mL of cyclohexane and 0.1 mL of dodecane (internal standard) were added in the reactor. Then, the reactor was closed and temperature of the mixture was increased to 140–150 °C under magnetic stirring. The pressure inside the reactor was stabilized at about 0.5 MPa. After 24 h, the temperature was cooled down to room temperature, and a sample (30 μL) was withdrawn from the reaction mixture, filtered through hydrophobic membrane, and injected into GC.

#### 3.3.2. Using Hydrogen Peroxide (H_2_O_2_)

The oxidation of cyclohexane over bulk NiO using H_2_O_2_ was performed as follows. Briefly, in a 50 mL flask equipped with a condenser, 50 mg of bulk NiO was dispersed in 10 mL of acetic acid, then 2 mL of the substrate (cyclohexane) and 0.1 mL of dodecane (internal reference) were added. After stirring this mixture for 5 min at 70 °C, 2.7 mL of H_2_O_2_ was added, wherein the 0 time of the reaction was considered at this moment. After 24 h, the organic phase was extracted by n-hexane and dried over MgSO_4_. Sample (30 μL) was withdrawn from the organic phase, filtered through hydrophobic membrane, and injected into GC.

#### 3.3.3. Tert-Butyl Hydroperoxide (TBHP)

The oxidation of cyclohexane over bulk NiO using TBHP was performed as follows. Briefly, in a 50 mL flask equipped with a condenser, the appropriate amount of bulk NiO (50 mg) was dispersed in 10 mL of acetonitrile, then 0.12 mL of substrate (cyclohexane) and 0.1 mL of dodecane (internal reference) were added. After stirring this mixture for 5 min at 70 °C, 0.3 mL of TBHP was added, wherein the 0 time of the reaction was considered at this moment. After 24 h, a sample of about 30 μL was withdrawn from the reaction mixture, filtered through hydrophobic membrane to remove the solid catalyst, and then injected into GC.

#### 3.3.4. *Meta*-Chloroperoxybenzoic Acid (*m*-CPBA)

The oxidation of cyclohexane over bulk NiO using *m*-CPBA was performed following a modified procedure reported by Abboud et al. [34]. Briefly, in a 50 mL flask equipped with a condenser, 50 mg of bulk NiO was dispersed in 10 mL of acetonitrile, then 0.12 mL of cyclohexane (substrate) and 0.1 mL of dodecane (internal reference) were added. After stirring this mixture for 5 min at 70 °C, 288 mg of *m*-CPBA was added, wherein the 0 time of the reaction was considered at this moment. After 24 h, a sample of about 30 μL was withdrawn from the reaction mixture, filtered through hydrophobic membrane to remove the solid catalyst, and then injected into GC.

#### 3.3.5. The Optimization of the Oxidation of Cyclohexane over Bulk NiO Using *m*-CPBA

Following the same procedure described in Section 3.4 above, the catalyst dose, temperature, and reaction time were studied, as described in the following Table 1.

### 3.4. The Calculation of Conversion and Selectivity

The conversion and selectivity were calculated according to the following equations:

(1) $$\mathrm{Conversion}\;\left(\%\right)=100-\frac{Peak\;area\;of\;cyclohexane}{Peak\;areas\;of\;\left(cyclohexane+all\;products\right)}\times 100$$

Equation (1). Conversion calculation.

(2) $$\mathrm{Selectivity}\;\mathrm{to}\;\mathrm{CXAON}\;\left(\%\right)=\frac{Peak\;area\;of\;cyclohexanone}{Peak\;areas\;of\;all\;products}\times 100$$

Equation (2). Selectivity to CXAON calculation.

(3) $$\mathrm{Selectivity}\;\mathrm{to}\;\mathrm{CXAOL}\;\left(\%\right)=\frac{Peak\;area\;of\;cyclohexanol}{Peak\;areas\;of\;all\;products}\times 100$$

Equation (3). Selectivity to CXAOL calculation.

### 3.5. Catalyst Recycling

The bulk NiO was reused in five successive cycles following the same procedure described in Section 3.4. After 24 h of the first cycle, the mixture was filtered using a centrifuge to recover the catalyst. The catalyst was washed three times with chloroform to remove the remaining substrate, products, internal reference, *m*-CPBA, solvent, and the *meta*-chlorobenzoic acid (*m*-CBA) formed after the degradation of *m*-CPBA. Then, the recycled catalyst was dried in the oven at 100 °C for around 15 h to be ready for the following cycle. At the end of each cycle, a sample of about 30 μL was withdrawn from the reaction mixture, filtered through hydrophobic membrane to remove the solid catalyst, and then injected into GC.

### 3.6. Possible Mechanism

*m*-CPBA is among the most important organic peroxides (ROOH or ROOR’) that have been used as strong oxidants for various organic transformations. *m*-CPBA showed high activity even for the hydroxylation reaction of methane in the presence of iron porphyrin complex or its enzymatic model P450 [48,49]. *m*-CPBA also has been used to activate the C–H bonds of hydrocarbons, which is one of the most challenging chemical reactions in recent chemistry [50,51,52]. In contrast to O_2_, H_2_O_2_ and TBHP, *m*-CPBA is a stable and selective oxidant, which are important features in organic synthesis. Moreover, *m*-CPBA can form selective and highly active intermediates in the presence of auxiliary reagents [53,54,55]. In addition, *m*-CPBA is an easy-to-handle and convenient terminal oxidant. However, *m*-CPBA may produce a broader mixture of radicals [56,57,58]. Therefore, the presence of a catalyst is necessary to activate the O–O bond in a suitable way, and to suppress the secondary reaction pathways.

**Figure 12:**
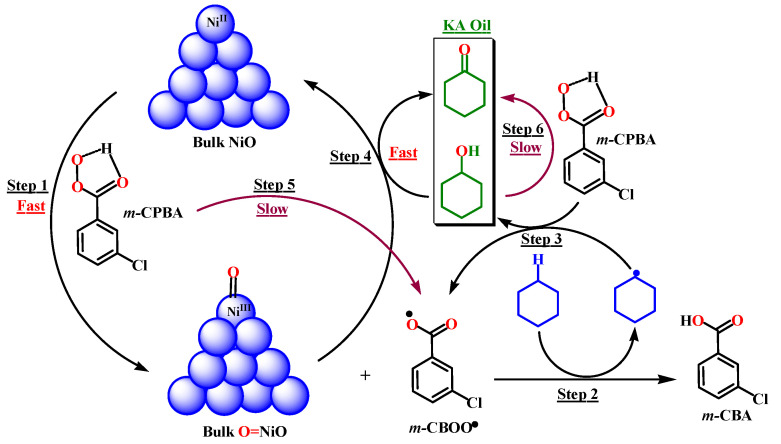
Possible mechanism for the oxidation of cyclohexane over bulk NiO.

According to some theoretical predictions and previous mechanistic investigations for the oxidation of hydrocarbons catalyzed by transition metals [38,59,60,61,62,63,64], and based on the obtained results in the current work, we believe that after homolysis of the O–O bond of *m*-CPBA in the presence of NiO active sites, nickel-oxo (O=NiO) species and *m*-CBOO• can be formed (Figure 12, step 1). In the proposed mechanism, *m*-CBOO• are the cyclohexane-C-H bond attacking species. After H abstraction by *m*-CBOO• species, cyclohexane radical (CXA•) can be formed with the generation of *m*-CBA (Figure 12, step 2). CXA• reacts with another molecule of *m*-CPBA to form CXAOL with the regeneration of *m*-CBOO• species (Figure 12, step 3). Then, a portion of CXAOL will be converted to CXAON after further oxidation over NiO (Figure 12, step 4). The ratio CXAON/CXAOL is related to the catalyst dose. The low conversion of CXA to CXAON and CXAOL over *m*-CPBA in the absence of the catalyst (Figure 10) can be explained by slow conversion of *m*-CPBA to *m*-CBOO• species under heat (Figure 12, step 5). In addition, the formation of a small amount of CXAON compared to CXAOL in the absence of the catalyst (Figure 10) can be attributed to the slow conversion of CXAOL to CXAON over *m*-CPBA (Figure 12, step 6).

## 4. Conclusions

This work reported an efficient process for the oxidation of cyclohexane to produce KA oil (cyclohexanone plus cyclohexanol) catalyzed by bulk NiO powder under mild conditions using *meta*-chloroperoxybenzoic acid (*m*-CPBA) as oxygen donor. NiO powder was prepared by simple calcination (500 °C, 5 h) of nickel (II) nitrate hexahydrate. For the first time, around 85% of cyclohexane was converted to products, with 99% KA oil selectivity, including about 87% of cyclohexanone (K) and 13% cyclohexanol (A), using 1.5 eq of *m*-CPBA as an oxidant, in acetonitrile as a solvent, and under 70 °C for 24 h. This reaction was monitored by gas chromatography (GC) using dodecane as internal standard. Furthermore, the reusability of NiO catalyst was also evaluated. This catalyst was easily separable and recyclable up to four cycles, with a slight decrease in the catalytic activity. After four cycles, the conversion of cyclohexane was decreased gradually to 63% with constant KA selectivity (99%), while the selectivity toward cyclohexanone was progressively decreased to 60%. Its features as a very efficient catalytic system under mild conditions, with low cost, easy synthesis, high cyclohexane conversion, high KA oil selectivity, and superiority to all reported catalysts available in the literature make this catalytic system very promising for the industrial production of adipic acid and nylon fibers.

## Figures and Tables

**Table 1:** Parameters investigated in the oxidation reaction of cyclohexane over bulk NiO using *m*-CPBA as oxygen donor.

Catalyst Dose (mg)	Temperature (°C)	Reaction Time (hours)
5, 10, 30, 50	25, 40, 60, 70	0.5, 1, 2, 4, 24

## Data Availability

Not applicable.
